# A Case Report of a Patient with Thyroid eye disease Induced by Hashimoto’s Thyroiditis

**DOI:** 10.14341/probl13597

**Published:** 2026-05-20

**Authors:** М. H. Khaskhazyan, D K. Shaghbatyan, T. A. Ambartsumyan, R. S. Mnatsakanyan, R. T. Shahbazyan

**Affiliations:** Medline clinic and National Institute of Health Named After Academician S. Avdalbekyan; Medline clinic and National Institute of Health Named After Academician S. Avdalbekyan; Ophthalmological center after S. V. Malayan; Ophthalmological center after S. V. Malayan; Yerevan State Medical University after Mkhitar Heratsi; Yerevan State Medical University after Mkhitar Heratsi; Erebuni medical center; Erebuni medical center; Yerevan State Medical University after Mkhitar Heratsi; Erebuni medical center

**Keywords:** Autoimmune thyroid disease, Hashimoto’s thyroiditis, Graves’ disease, Thyroid eye disease, Autoimmune thyroid disease, Hashimoto’s thyroiditis, Graves’ disease, Thyroid eye disease

## Abstract

**BACKGROUND:**

BACKGROUND: Autoimmune thyroid diseases (AITD) result from immune system dysregulation. Among them are Hashimoto’s thyroiditis, Graves’ disease, etc. Approximately 3% of patients with Hashimoto’s thyroiditis may develop orbitopathy. Thyroid eye disease (TED), is an organ-specific autoimmune reaction that primarily affects the eyes and surrounding tissues. The aim of this case report is to present a rare condition of a female patient who was diagnosed with exophthalmos nine years after being diagnosed with Hashimoto’s thyroiditis.

**CASE REPORT:**

CASE REPORT: A 45-year-old woman presented to our department with Hashimoto’s thyroiditis and hypothyroidism. Her medical history revealed that she was diagnosed with HT nine years ago and had been receiving L-thyroxine treatment throughout this period. However, her condition remained uncompensated, and in the past year, she developed bilateral exophthalmos as a complication.

**CONCLUSION:**

CONCLUSION: Exophthalmos is generally more strongly associated with Graves’ disease, a form of hyperthyroidism. However, it can also occur in some patients with Hashimoto’s thyroiditis, although it is less common.

## INTRODUCTION

Autoimmune thyroid diseases (AITD) are organ-specific autoimmune disorders. AITD includes interrelated conditions such as Hashimoto’s thyroiditis (HT), Graves’ disease (GD), atrophic autoimmune hypothyroidism, and postpartum thyroiditis, with HT and GD being the most prevalent. AITD is associated with two main types of antibodies: [1]

Thyroid eye disease is an autoimmune disease that occurs in patients with:

Thyroid ophthalmopathy involves an organ-specific autoimmune reaction where antibodies targeting thyroid gland cells and orbital fibroblasts lead to inflammation of extraocular muscles, interstitial tissues, orbital fat, and lacrimal glands. This inflammation is characterized by pleomorphic cellular infiltration, increased secretion of glycosaminoglycans, and water retention, which contribute to swelling, congestion, and connective tissue remodeling. As a result, patients develop extraocular muscle enlargement and orbital fat expansion [4].

## CASE REPORT

A 45-year-old female patient presented with complaints of:

Her medical history revealed that she had been diagnosed with Hashimoto’s thyroiditis and hypothyroidism since 2015. The patient was overweight, with a body mass index (BMI) of 28.5 kg/m². Over the past two years, she experienced ocular heaviness, grittiness, and excessive tearing (lacrimation).

## Laboratory Findings and Treatment History

In 2015, initial blood tests revealed hormonal imbalances:

She was prescribed L-thyroxine at a dose of 75 mcg/day. Due to symptom improvement, she did not undergo further medical follow-ups for several years.

In 2021, she was re-evaluated due to menstrual irregularities:

Her L-thyroxine dose was increased to 100 mcg/day.

In 2022, her TSH level remained elevated:

The dose of L-thyroxine was further increased to 175 mcg/day.

After one year on 175 mcg, the patient independently reduced her dose to 100 mcg/day, which led to the recurrence of symptoms, including eye protrusion (Fig. 1).

**Figure:**
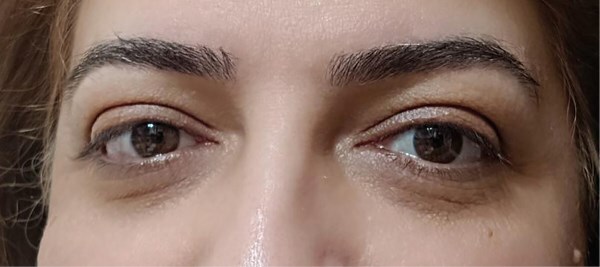
Figure 1. Patient with mild eye protrusion and dry eye syndrome.

In 2024, repeat testing showed:

Upon detailed history-taking, it was discovered that she had been incorrectly taking her medication by drinking coffee within 30–40 minutes of ingestion, reducing its effectiveness.

The L-thyroxine dose was increased to 200 mcg/day for two months, after which lab results improved:

However, she experienced palpitations, so the dose was adjusted to 175/150 mcg/day alternately. This adjustment restored her menstrual cycle and resolved the palpitations.

Three months later, her hormone levels stabilized:

## Ophthalmological Evaluation and Management

The patient presented with mild eye protrusion, as classified by the European Group on Graves’ Orbitopathy (EUGOGO), and dry eye syndrome. This case highlights the importance of recognizing this uncommon manifestation [3].

Ophthalmological findings:

**Figure:**
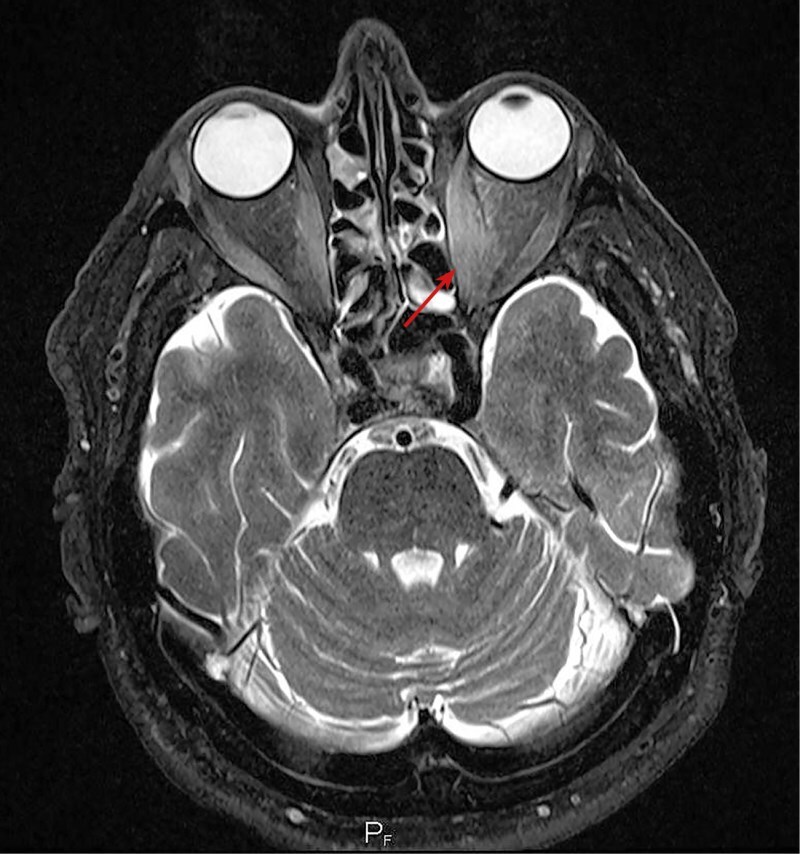
Figure 2. MRI scan showing muscle enlargement without compressing the optic nerve.

## Treatment and Outcome

Management of mild ophthalmopathy included:

Follow-up exophthalmometry showed slight regression of the eye prominence, with near-normal measurements.

## CONCLUSION

Thyroid eye disease is an autoimmune inflammatory disorder characterized by distinct clinical features. While exophthalmos is more commonly associated with Graves’ disease, it can also occur in Hashimoto’s thyroiditis, although rarely.

This case highlights the importance of properly managing chronic hypothyroidism and ensuring correct medication intake. It is crucial to maintain an appropriate time gap between taking thyroid medication and consuming food or beverages to ensure proper absorption and effectiveness.

## ADDITIONAL INFORMATION

Funding sources. This work was carried out on the authors’ initiative without external funding.

Conflict of interest. The authors declare no actual or potential conflicts of interest related to the content of this article.

Author contributions. All authors approved the final version of the article prior to publication and agreed to be accountable for all aspects of the work, including ensuring that questions related to the accuracy or integrity of any part of the work are appropriately investigated and resolved.
